# Pulsed Field Ablation Versus High-Power Short-Duration Ablation for Atrial Fibrillation—A Meta-Analysis of Reconstructed Time-to-Event Data

**DOI:** 10.3390/jcdd13050181

**Published:** 2026-04-26

**Authors:** Pedro B. Bregion, Felipe S. Passos, Stefano Schena, Luca Fazzini, Hristo Kirov, Camilla S. Rossi, Torsten Doenst, Antonino Di Franco, Tulio Caldonazo

**Affiliations:** 1 Department of Medicine, State University of Campinas, Campinas 13083-970, Brazil; 2 Department of Thoracic Surgery, MaterDei Hospital, Salvador 40220-005, Brazil; felipesanpassoss@gmail.com; 3 Division of Cardiac Surgery, Medical College of Wisconsin, Milwaukee, WI 53226, USA; 4 Clinical Cardiology Unit, Department of Medical Sciences and Public Health, University of Cagliari, 09042 Cagliari, Italy; 5 Department of Cardiothoracic Surgery, Friedrich-Schiller-University Jena, 07747 Jena, Germany; 6 Department of Cardiothoracic Surgery, Weill Cornell Medicine, New York, NY 10065, USA

**Keywords:** atrial fibrillation, pulsed field ablation, high-power short duration

## Abstract

Background: Pulsed field ablation (PFA) has shown promising results for atrial fibrillation (AF), with efficacy comparable to established ablation techniques. High-power short-duration (HPSD) ablation has also emerged as a potential alternative. However, the relative superiority between these approaches remains uncertain. We performed a systematic review and meta-analysis to address this gap. Methods: Three databases were searched. The primary outcome was freedom from AF recurrence. Secondary outcomes included tamponade and other complications, procedure and fluoroscopy durations. Time-to-event data were reconstructed, and a random-effects model was employed. Given variability in post-ablation blanking periods across studies, landmark analyses were performed using a 3-month cut-off to account for the potential under-detection of early recurrence events. Results: Eight studies (1369 patients [PFA: 642; HPSD: 727]) were included. PFA was associated with greater freedom from AF recurrence (HR 0.751; 95% CI 0.57 to 0.99; *p* = 0.044). Landmark analyses showed no difference in the 0–3 month period; a significant benefit of PFA was observed thereafter (HR 0.72; 95% CI 0.54 to 0.98; *p* = 0.033). There were no significant differences between groups in the incidence of tamponade (*p* = 0.73) or overall complications (*p* = 0.99). PFA was associated with shorter procedure duration (MD 37.05; 95% CI 27.69 to 46.41; *p* < 0.01), whereas fluoroscopy duration was significantly shorter in the HPSD group (MD −9.04; 95% CI −11.71 to −6.37; *p* < 0.001). Conclusion: PFA was associated with a lower risk of AF recurrence compared to HPSD, particularly beyond the late post-ablation period, with similar rates of complications. Although PFA was associated with shorter procedure duration, HPSD demonstrated reduced fluoroscopy time.

## 1. Introduction

Pulmonary vein isolation (PVI) is the gold standard for catheter ablation in patients with symptomatic paroxysmal or persistent atrial fibrillation (PAF and persAF) [1]. The most commonly adopted approaches to achieve PVI are cryoballoon (CB) and radiofrequency (RF) catheter ablation [1,2,3]. Despite their effectiveness, limitations regarding procedural safety and durability of lesion sets have driven the development of novel energy sources and delivery strategies to improve outcomes. Among these, pulsed field ablation (PFA) and high-power short-duration (HPSD) RF ablation have recently emerged as promising alternatives.

PFA delivers high-amplitude electrical fields in ultra-short pulses, inducing irreversible electroporation of myocytes [4,5]. Unlike thermal energy, this nonthermal mechanism minimizes collateral tissue damage, particularly to structures adjacent to the pulmonary veins, such as the esophagus and the phrenic nerve [6,7]. Clinical studies have shown that PFA achieves comparable efficacy to CB and RF ablation, while reducing the risks of pulmonary vein stenosis and extracardiac injury [1,7,8,9,10]. Reported arrhythmia-free survival rates at one year range from 70% to 85% across different PFA techniques, with shorter procedure times and a favorable safety profile [7,11,12,13].

In parallel, HPSD RF ablation represents a refinement of traditional low-power long-duration (LPLD) strategies. While conventional LPLD settings employ 30 W for 30 s [14,15,16], HPSD typically applies >45 W for <20 s, with very-high-power short-duration (VHPSD) protocols reaching 70 W for 5 s. These approaches have been associated with shorter procedures, improved lesion durability, and superior long-term outcomes compared with LPLD [17,18,19,20]. However, although several recent meta-analyses have compared PFA and HPSD/VHPSD strategies, important uncertainties remain regarding the time-dependent pattern of recurrence and the interpretation of pooled results across heterogeneous energy settings. Therefore, we conducted a meta-analysis focused on reconstructed individual patient time-to-event data, aiming to provide a more granular evaluation of AF recurrence dynamics while also comparing procedural performance and safety outcomes between PFA and HPSD for pulmonary vein isolation.

## 2. Methods

This systematic review and meta-analysis were conducted according to the Cochrane Collaboration Handbook for Systematic Reviews of Interventions and Preferred Reporting Items for Systematic Reviews and Meta-Analyses (PRISMA) guidelines statement. The complete PRISMA checklist is presented in Supplementary Table S1. It did not require ethical approval as no human or animal subjects were involved. This review was registered with the National Institute for Health Research International Registry of Systematic Reviews (PROSPERO; CRD42024553374).

### 2.1. Search Strategy

A comprehensive literature search was performed on PubMed, Scopus and Cochrane Library to identify contemporary studies comparing PFA and HPSD in patients with AF from inception to March 2026. The complete search strategy is available in Supplementary Table S2.

### 2.2. Study Selection

Two independent reviewers (PB and LF) screened the records after de-duplication. Any discrepancies and disagreements were resolved by a third author (TC). Titles and abstracts were reviewed against pre-defined inclusion and exclusion criteria.

### 2.3. Eligibility Criteria and Quality Assessment

Inclusion criteria were: (1) observational studies, randomized controlled trials (RCTs), or abstracts; (2) published in English; (3) comparing outcomes in patients with PAF or persAF treated with PFA versus HPSD; and (4) reporting registry-based results on AF recurrence or procedural characteristics. Included studies encompassed PAF [13,21], persAF [22] or mixed populations [23,24,25], though outcomes were not always reported separately. One study noted 56% PAF without specifying the remainder. Exclusion criteria were non-English articles, studies without relevant outcomes, case reports, and non-comparative designs. For the purpose of study selection, HPSD was defined as radiofrequency ablation delivered at 45–50 W, whereas VHPSD referred to protocols using 70–90 W. Because several included studies reported these approaches under a broader HPSD framework, both strategies were retained for the main analysis, but this variability was considered a potential source of heterogeneity and is explicitly addressed in the Discussion. References of included articles were screened for additional studies. Study quality was assessed with the Newcastle–Ottawa Scale (NOS). Publication bias could not be assessed due to the limited number of included studies.

### 2.4. Data Extraction

Two reviewers (PB and LF) independently performed data extraction. Accuracy was verified by a third author (TC). The extracted variables included study characteristics (publication year, sample size, study design, follow-up, type of PFA catheter, type of HPSD catheter, blanking period duration, and energy used on the procedure) as well as patient demographics (age, sex, body mass index (BMI), hypertension, diabetes mellitus, mean left ventricular ejection fraction (LVEF), number of patients with PAF and persAF).

### 2.5. Outcomes

The primary outcome was freedom from AF recurrence. Secondary outcomes were tamponade and overall complications, procedure duration and fluoroscopy duration.

### 2.6. Statistical Analysis

Odds ratio (OR) and Mean Difference (MD) with 95% confidence intervals (CIs) were calculated for binary and continuous outcomes, respectively. The reconstructed time-to-event data strategy was applied for the primary outcome. A random-effects model was used to account for clinical heterogeneity across studies. Heterogeneity was assessed using the Cochran Q test and I^2^ statistic, with *p* < 0.10 and I^2^ > 50% indicating significant heterogeneity. A complementary two-stage meta-analysis was conducted for the primary outcome. For outcomes with high heterogeneity, a leave-one-out sensitivity analysis was performed. When means and standard deviations were not reported, they were estimated from medians and ranges using the method described by Wan et al. [26]. A subgroup analysis according to AF subtype (paroxysmal versus persistent) was not feasible because most of the included studies reported mixed populations and did not provide outcome data separately for each AF subtype.

### 2.7. Individual Patient AF Recurrence Data Meta-Analysis

For all eligible studies for freedom from AF recurrence, we used the methods described by Wei et al. [27,28] to reconstruct individual patient data (IPD) with the Kaplan–Meier estimator. Raster and Vector images of the Kaplan–Meier survival curves were pre-processed and digitized, so that the values corresponding to specific timepoints with their corresponding freedom-from-recurrence probabilities information could be extracted. To calibrate the accuracy of this time to event, we used additional information (e.g., number-at-risk tables or total number of events). To confirm the quality of the timing of failure events captured, we thoroughly checked the consistency with the reported survival or mortality data provided in the original publications.

### 2.8. Meta-Analysis of Reconstructed Data—One-Stage AF Recurrence Meta-Analysis

The Kaplan–Meier method was used to calculate the overall freedom from AF recurrence. The Cox proportional hazards regression model was used to assess between-group differences. For these Cox models, the proportional hazards assumption was verified by plotting scaled Schoenfeld residuals, log–log survival plots, and predicted versus observed survival functions. Given the variability in the definition and use of post-ablation blanking periods across studies (most common duration: three months), and the potential under-detection of early recurrences during this phase, we performed a landmark analysis using a three-month cut-off. We plotted survival curves using the Kaplan–Meier product limit method and calculated the hazard ratios (HRs) and 95% CIs of each group. All statistical analyses were performed using R (version 4.4.0, R Project for Statistical Computing) and STATA 17.0 (StataCorp LLC, College Station, TX, USA).

## 3. Results

### 3.1. Study Characteristics

**Figure 1:**
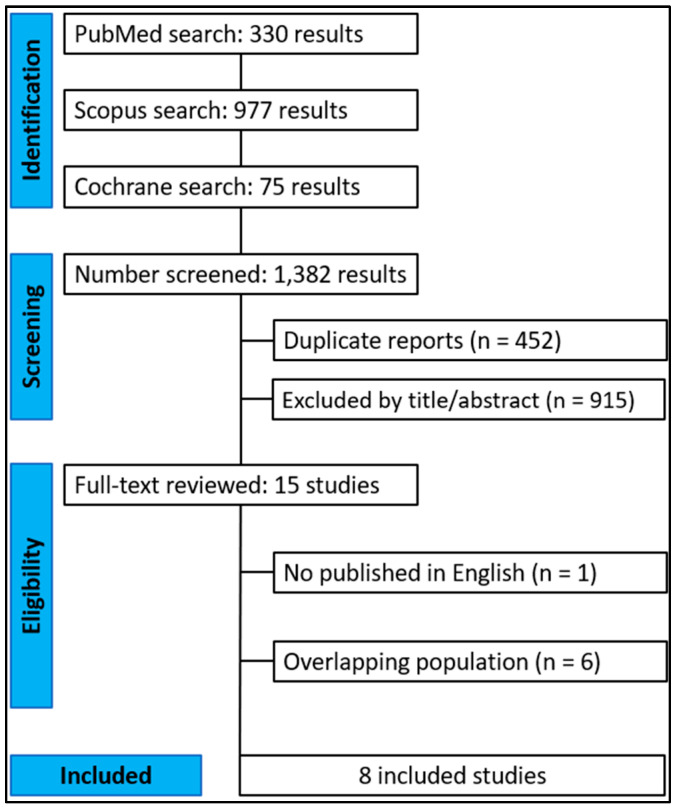
Preferred Reporting Items for Systematic Reviews and Meta-Analyses (PRISMA) flow diagram.

Figure 1 shows the PRISMA flowchart for study selection. The initial search retrieved a total of 1382 studies, of which eight [13,21,22,23,24,25,29,30] met all the eligibility criteria for inclusion in the final analysis. Included studies were published between 2023 and 2024. Detailed study characteristics are presented in Table 1.

### 3.2. Patient Characteristics

When available, propensity-scored matched cohorts were considered. Therefore, the final analysis included a total of 1369 patients [PFA: 642; HPSD: 727] from eight observational studies, with sample sizes ranging from 79 to 410. Demographic details of each study are summarized in Supplementary Table S3. The mean age of participants ranged from 61.6 to 73 years. The power settings for HPSD ablation ranged from 45 W to 90 W.

In terms of procedural tools, the PFA group primarily employed the Farapulse™ system, utilizing the Farawave™ pentaspline catheter and the Farastar™ generator. Sheath selection for PFA included the Faradrive™ steerable sheath and the Swartz™ Braided LAMP™ 45. The HPSD/vHPSD group used a variety of advanced irrigated-tip catheters, most notably the QDOT Micro™ (operating in temperature-controlled QMODE+), the FlexAbility™ SE, and the Thermocool SmartTouch SF^®^. These procedures were supported by 3D electroanatomical mapping systems, including CARTO^®^ 3 and EnSite™ Precision/X. Thermal ablation sheath selection included the Agilis™ steerable sheath, the CARTO VIZIGO™ (often used for 90 W procedures), and the TSX™ or SL1 Fast-Cath sheaths.

### 3.3. Quality Assessment

The risk of bias is presented in Supplementary Table S4. Overall, most studies demonstrated moderate methodological quality according to the NOS. Selection domains were generally well addressed, with five studies achieving the maximum score of four stars, two studies receiving three stars, and one study receiving two stars. Comparability varied across studies: four studies achieved two stars, indicating adequate adjustment for confounders, whereas the remaining studies demonstrated limited adjustment and received only one star. Outcome/exposure domains were adequately reported in most studies, with three achieving the maximum score of three stars, three receiving two stars, and two studies showing more limited outcome assessment or follow-up adequacy, receiving one star. Overall, the studies showed moderate methodological quality, although the limited comparability across studies suggests a potential risk of residual confounding that should be considered when interpreting the results.

### 3.4. Primary Outcome—Freedom from AF Recurrence

**Figure 2:**
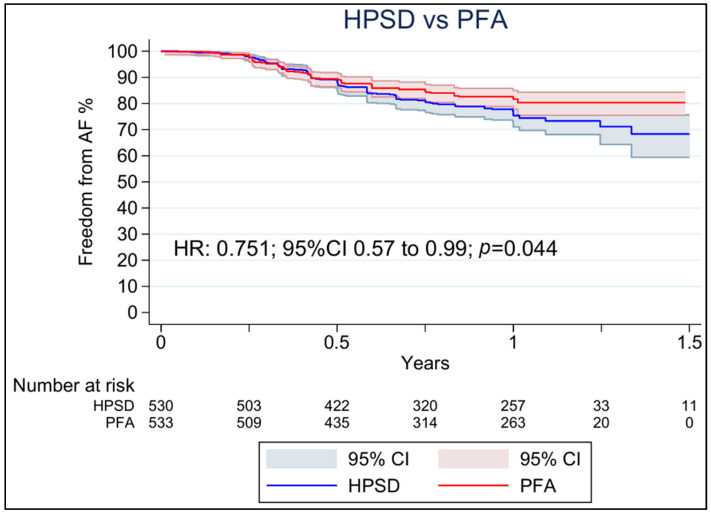
Pooled Kaplan–Meier curves showing long-term freedom from AF recurrence following PFA and HPSD. CI: confidence interval, HPSD: high-power short duration, HR: hazard ratio, and PFA: pulsed field ablation.

Table 2 summarizes the main findings of this meta-analysis. For freedom from AF recurrence, five studies reported this outcome using Kaplan–Meier curves, which were processed, digitized, and reconstructed. Post-ablation blanking periods varied across studies, ranging from no formal blanking period [21] to durations of one [24] and three months [13,23,30], which may have influenced the capture of early recurrent events. Figure 2 shows the pooled Kaplan–Meier curves for the entire observation period. Overall, PFA was associated with greater freedom from AF recurrence compared with HPSD (HR 0.751; 95% CI 0.57 to 0.99; *p* = 0.044; Figure 2).

### 3.5. Sensitivity Analyses

**Figure 3:**
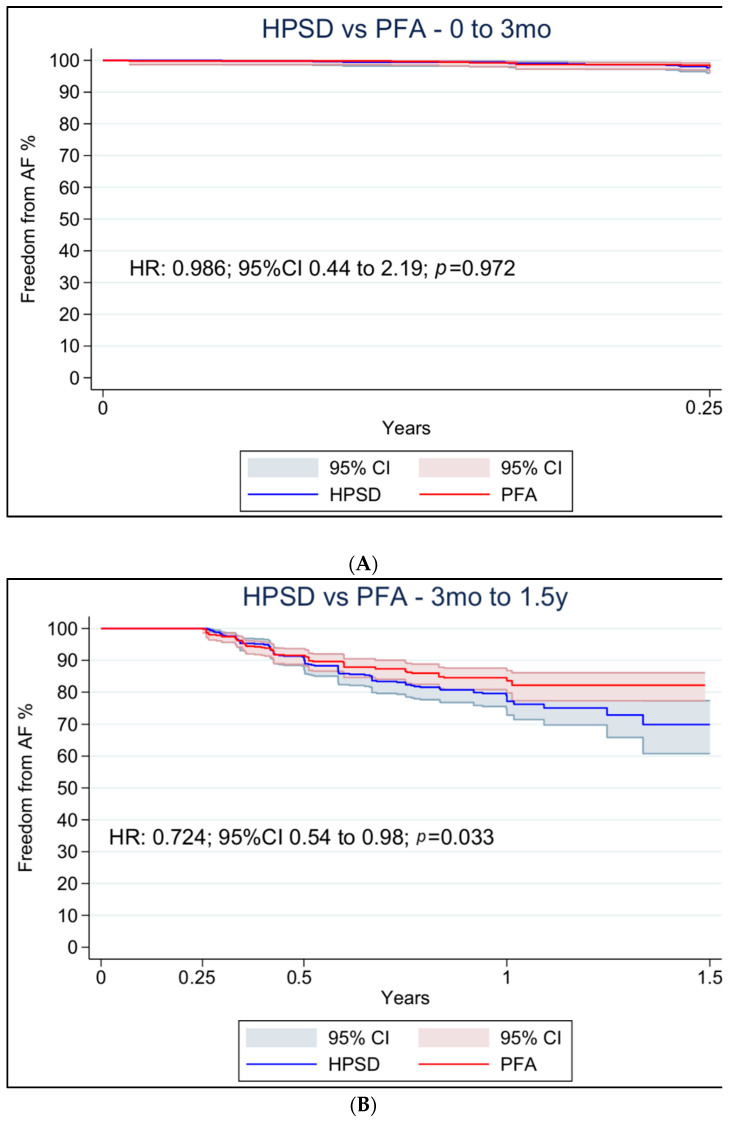
Landmark analysis comparing freedom from AF recurrence between PFA and HPSD ablation. (**A**) 0–3 months post-ablation; (**B**) 3 months to 1.5 years post-ablation. CI: confidence interval, HPSD: high-power short duration, HR: hazard ratio, and PFA: pulsed field ablation.

Violations of the proportional hazards assumption were identified for freedom from AF (Supplementary Figure S1). Accordingly, separate Cox models were applied for two time intervals (0–3 months and 3 months to 1.5 years), with the 3-month cut-off reflecting the most commonly adopted blanking period across studies. No difference was observed in the early period (HR 0.99; 95% CI 0.44 to 2.19; *p* = 0.97; Figure 3A), whereas PFA was associated with a lower risk of recurrence in the later period (HR 0.72; 95% CI 0.54 to 0.98; *p* = 0.033; Figure 3B).

A complementary two-stage meta-analysis, performed as an additional approach to support the reconstructed Kaplan–Meier analysis, confirmed these findings, showing a significant reduction in AF recurrence with PFA (HR 0.75; 95% CI 0.56 to 0.99; *p* = 0.04; I^2^ = 0%; Supplementary Figure S2).

### 3.6. Secondary Outcomes

**Figure 4:**
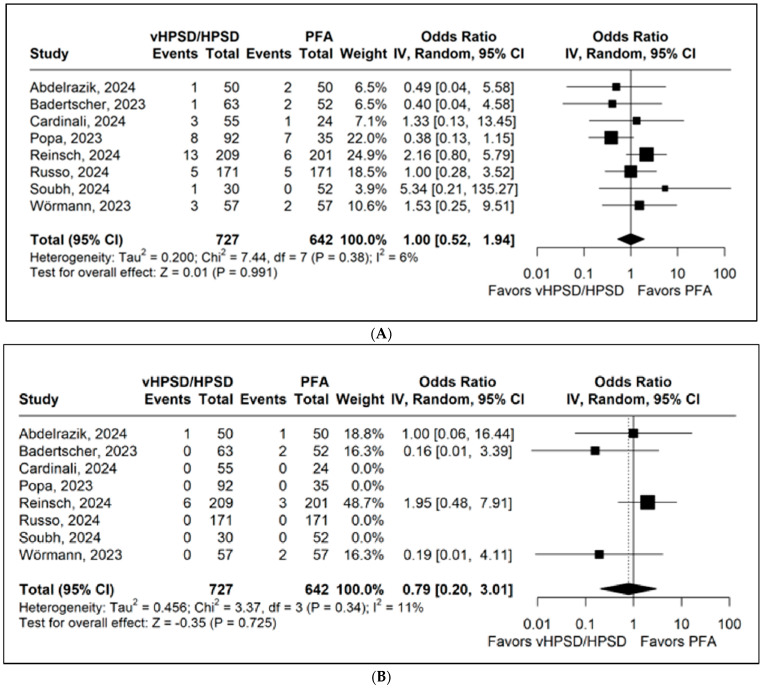
Forest plots comparing the incidence of (**A**) overall complications and (**B**) tamponade between PFA and HPSD ablation. CI: confidence interval, HPSD: high-power short duration, OR: odds ratio, and PFA: pulsed field ablation. Citations: Abdelrazik [25]; Badertscher [21]; Cardinali [29]; Popa [22]; Reinsch [13]; Russo [24]; Soubh [30]; and Wörmann [23].

Eight studies, encompassing 1369 patients, reported overall complications. The pooled analysis did not demonstrate a statistically significant difference between groups (OR 1.00; 95% CI 0.52 to 1.94; *p* = 0.991; I^2^ = 6%; Figure 4A).

Eight studies, including 1369 patients, reported cardiac tamponade. The pooled analysis showed no significant difference between HPSD and PFA (OR 0.79; 95% CI 0.20 to 3.01; *p* = 0.725; I^2^ = 11%; Figure 4B).

**Figure 5:**
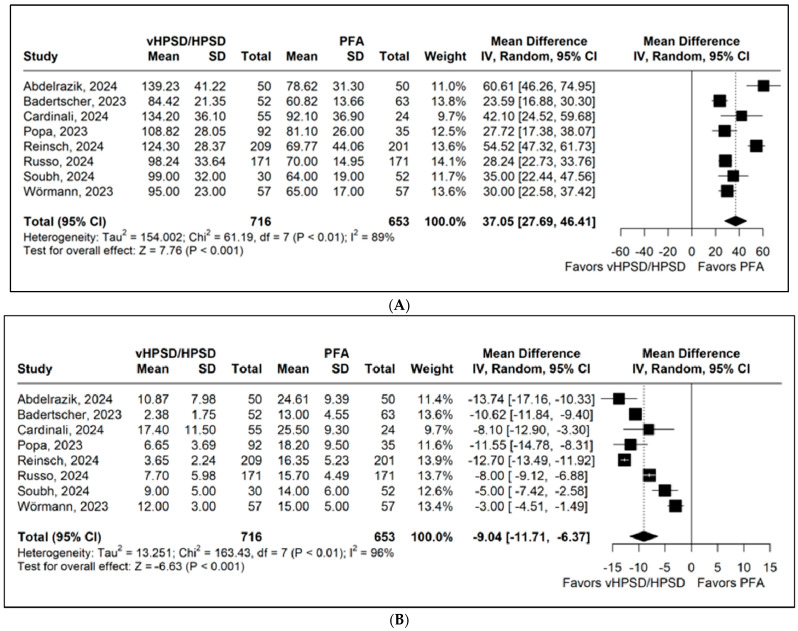
Forest plots comparing (**A**) procedure duration and (**B**) fluoroscopy duration between PFA and HPSD ablation. CI: confidence interval, HPSD: high-power short duration, MD: mean difference, and PFA: pulsed field ablation. Citations: Abdelrazik [25]; Badertscher [21]; Cardinali [29]; Popa [22]; Reinsch [13]; Russo [24]; Soubh [30]; and Wörmann [23].

Eight studies, comprising 1369 patients, reported procedure duration. The pooled analysis demonstrated significantly shorter procedure times in the PFA group (MD 37.05 min; 95% CI 27.69 to 46.41; *p* < 0.001; I^2^ = 89%; Figure 5A).

Eight studies, including 1369 patients, reported fluoroscopy duration. The pooled analysis showed significantly shorter fluoroscopy time in the HPSD group (MD −9.04 min; 95% CI −11.71 to −6.37; *p* < 0.001; I^2^ = 96%; Figure 5B).

### 3.7. Sensitivity Analysis

To assess the robustness of these findings, a leave-one-out sensitivity analysis was performed for outcomes with high heterogeneity. For procedure and fluoroscopy duration, the overall direction and magnitude of the effect remained consistent after sequential exclusion of individual studies (Supplementary Figures S3 and S4), supporting the stability of these results.

Assessment of publication bias was not performed for tamponade and overall complications due to the limited number of included studies, which precludes reliable interpretation of funnel plots and renders statistical tests such as Egger’s test inappropriate.

## 4. Discussion

In this systematic review and meta-analysis of eight studies including 1369 patients with PAF and persAF, we compared the safety and efficacy of PFA and HPSD ablation. Our main findings were as follows: (I) PFA was associated with an improved freedom from AF recurrence, particularly beyond the late post-ablation period, likely reflecting the limited capture of events during the blanking period rather than a true absence of early differences; (II) there were no significant differences in overall complications or tamponade; (III) PFA was associated with shorter procedure duration, with substantial heterogeneity; and (IV) fluoroscopy duration was significantly shorter in the HPSD group, also with considerable heterogeneity.

PFA and HPSD are different ablation strategies that have shown promising results in the treatment of AF [4,6,19,20]. While PFA uses nonthermal electroporation to achieve selective myocardial injury, HPSD represents an evolution of RF ablation by delivering higher power over shorter durations [31,32,33,34]. In our analysis, both approaches demonstrated similar safety profiles, with PFA associated with shorter procedure times, suggesting potential gains in efficiency. These findings are consistent with recent meta-analyses. Mariani et al. [35] reported lower AF recurrence with PFA, while Pranata et al. [36] and Amin et al. [37] also described improved procedural efficiency and a possible reduction in recurrence, although this advantage appears less evident when compared specifically with VHPSD strategies.

Using a reconstructed IPD time-to-event approach, we identified a time-dependent effect. While no difference was observed in the early post-ablation period, this finding should be interpreted with caution, as most included studies applied a blanking period of up to three months, during which arrhythmia recurrences were not systematically captured or considered as treatment failure. When accounting for this, landmark analysis demonstrated that the benefit of PFA emerged beyond three months, with a lower risk of AF recurrence in the later phase. This finding suggests that a single global model assuming proportional hazards may incompletely capture the time-dependent dynamics of AF recurrence in this setting.

The safety profiles of PFA and HPSD were also found to be comparable in our analysis, with no significant differences in complication rates, including tamponade. This finding aligns with previous literature and suggests that both techniques offer similar safety outcomes [10,11,19,20]. The evolution of HPSD, particularly with the integration of CF-sensing technology, appears to mitigate some of the risks traditionally associated with RF ablation, such as thrombus formation and esophageal injury [16,32,33]. Meanwhile, the shorter procedure time observed with PFA may support its consideration in clinical settings where time efficiency is critical. However, these findings should be interpreted with caution, given the substantial heterogeneity observed in our analysis. This variability likely reflects differences across studies, including catheter platforms, operator experience, mapping systems, and AF subtype distribution. In addition, the inclusion of both conventional HPSD and VHPSD strategies within the same analytical framework may have further contributed to this inconsistency.

Our results are consistent with prior trials and registries, including IMPULSE, PEFCAT, and MANIFEST-PF, which have demonstrated favorable efficacy and safety profiles for PFA compared with thermal ablation techniques [4,11,12]. In this context, the comparable outcomes observed between PFA and HPSD in our study further support the role of both strategies as viable alternatives in contemporary AF ablation. Importantly, given the observational nature of the available evidence, these findings should be interpreted as hypothesis-generating.

Beyond traditional outcomes, the management of AF is increasingly focusing on patient-reported outcomes (PROMs), such as quality of life and symptom burden. Although our results show similar efficacy and safety between PFA and HPSD, these outcomes may not fully capture how patients actually feel during recovery. Recent evidence, including the study by Matteucci et al. [38], suggests that PFA may be associated with better quality of life and faster functional recovery, possibly due to its non-thermal mechanism and reduced collateral tissue injury. Together, these findings reinforce the importance of considering PROMs in both future research and clinical decision-making.

Our study addresses a critical gap in the literature by employing a meta-analysis of reconstructed time-to-event data, allowing for a more precise estimation of long-term AF recurrence. This methodological approach provides a robust framework for comparing PFA and HPSD, particularly in the absence of large-scale randomized controlled trials directly comparing these two techniques. Importantly, by incorporating landmark analysis aligned with the most commonly used blanking period, we accounted for the likely under-detection of early recurrences. This suggests that the absence of a difference in the early phase may reflect limited event capture rather than a true lack of biological effect. Moreover, the shorter procedure duration observed with PFA in our analysis offers additional value for clinical decision-making, where minimizing procedure duration without compromising efficacy is a priority. By integrating individual patient data (IPD) from Kaplan–Meier curves, our analysis offers deeper insights into time-to-event outcomes, crucial for evaluating the long-term efficacy of ablation strategies.

This meta-analysis has several limitations that may affect the generalizability of our findings. Only eight studies were included, with the majority being retrospective and only one prospective study, and no randomized comparisons were available. The included populations were heterogeneous, with different follow-up protocols and relatively short and variable follow-up durations (approximately 2 to 26 months), which may have influenced recurrence estimates. Additionally, differences in the definition and application of post-ablation blanking periods across studies may have contributed to heterogeneity, particularly in the assessment of early recurrence events. Subgroup analyses according to AF subtype were not feasible, as most studies reported mixed populations without stratified outcomes, despite known differences in recurrence patterns between paroxysmal and persistent AF. In addition, variability in procedural aspects, such as catheter types, ablation techniques, and energy settings, likely contributed to the observed heterogeneity. Although no clear publication bias was detected, it cannot be excluded given the small number of studies, and formal tests such as Egger’s test could not be performed. Despite these limitations, our findings suggest that PFA may be associated with lower late AF recurrence, with broadly similar complication rates and shorter procedure duration; however, these findings remain exploratory and require confirmation in randomized studies.

## 5. Conclusions

PFA was associated with a lower risk of AF recurrence compared to HPSD, particularly beyond the late post-ablation period, with similar rates of complications. Although PFA was associated with shorter procedure duration, HPSD demonstrated reduced fluoroscopy time. These findings should be interpreted as hypothesis-generating and require confirmation in future randomized studies.

## Figures and Tables

**Table 1:** Summary of included articles.

Author	Sample Size, HPSD/PFA, n	Type of Study	Kind of Patients	Follow-Up, Min/ Max (Months)	Median Follow-Up (Months)	Blanking Period Duration (Months)	Type of PFA Catheter	Type of HPSD Catheter	HPSD Energy (W)
Abdelrazik, 2024 [25]	50/50	Retrospective	Parox./Pers.	NR	NR	NR	Farapulse™	QDOT Micro™ (QMODE+)	90 W
Badertscher, 2023 [21]	63/52	Prospective	Paroxysmal	3.5/12.6	7	NR	Farawave™	Thermocool SmartTouch SF	50 W
Cardinali, 2024 [29]	55/24	Retrospective	Persistent	9/26	14	NR	Farapulse™ (implied)	QDOT Micro™ (implied)	90 W (Post)/ 50 W (Ant)
Popa, 2023 [22]	92/35	Retrospective	Paroxysmal	3/6	NR	2	Farawave™	FlexAbility™ SE/QDOT Micro™	70 W/ 90 W
Reinsch, 2024 [13]	209/201	Retrospective	Paroxysmal	3/12	12	3	Farawave™	Thermocool SmartTouch SF	45 W
Russo, 2024 [24]	171/171 *	Retrospective	Parox./Pers.	9/12	12	1	Farawave™	QDOT Micro™	90 W (QMODE+)
Soubh, 2024 [30]	30/52	Retrospective	Parox./Pers.	6/6	6	3	Farawave™	QDOT Micro™	90 W (QMODE+)
Wörmann, 2023 [23]	57/57	Retrospective	Parox./Pers.	3.6/5.4	4.1	3	Farawave™	FlexAbility™	70 W (7 s/5 s)

HPSD: high-power short-duration; NR: not reported; PFA: pulsed field ablation; * Propensity score–matched population; values refer to the matched cohort.

**Table 2:** Outcomes summary.

Outcome	Number of Studies	Number of Patients	Effect Estimate (95% CI, *p*-Value)
Freedom from AF recurrence	5	1063	HR 0.751; 95% CI 0.57 to 0.99; *p* = 0.044
Tamponade	8	1369	OR 0.79; 95% CI 0.20 to 3.01; *p* = 0.73; I^2^ = 11%
Overall complications	8	1369	OR 1.00; 95% CI 0.52 to 1.94; *p* = 0.991; I^2^ = 6%
Procedure duration	8	1369	MD 37.05; 95% CI 27.69 to 46.41; *p* < 0.01; I^2^ = 89%
Fluoroscopy duration	8	1369	MD −9.04; 95% CI −11.71 to −6.37; *p* < 0.001; I^2^ = 96%

AF = atrial fibrillation; CI = confidence interval; HR = hazard ratio; MD = mean difference; and OR = odds ratio.

## Data Availability

The original contributions presented in this study are included in the article/Supplementary Materials. Further inquiries can be directed to the corresponding author.

## Supplementary Materials

The following supporting information can be downloaded at: https://www.mdpi.com/article/10.3390/jcdd13050181/s1, Table S1. PRISMA checklist; Table S2. Search strategy for Ovid MEDLINE; Table S3. Demographics of included patients from the selected studies; Table S4. Assessment of risk of bias using the Newcastle Ottawa Scale; Figure S1. Schoenfeld residuals plot assessing the proportional hazards assumption for freedom from AF recurrence; Figure S2. Two-stage random-effects meta-analysis of freedom from AF recurrence; Figure S3. Leave-one-out analysis for procedure duration; Figure S4. Leave-one-out analysis for fluoroscopy duration.
