# Supplementary material for: Pulsed Field Ablation Versus High-Power Short-Duration Ablation for Atrial Fibrillation—A Meta-Analysis of Reconstructed Time-to-Event Data
Source: J Cardiovasc Dev Dis. 2026 Apr 26;13(5):181. doi: 10.3390/jcdd13050181 (PMC13207704; doi:10.3390/jcdd13050181)

Supplementary Material

Table S1. PRISMA checklist

Table S2. Search strategy for Ovid MEDLINE.

Table S3. Demographics of included patients from the selected studies.

Table S4. Assessment of risk of bias using the Newcastle Ottawa Scale.

Figure S1. Schoenfeld residuals plot assessing the proportional hazards assumption for freedom from AF recurrence

Figure S2. Two-stage random-effects meta-analysis of freedom from AF recurrence

Figure S3. Leave-one-out analysis for procedure duration.

Figure S4. Leave-one-out analysis for fluoroscopy duration.

Table S1. PRISMA checklist

Page Section and Topic	Item #	Checklist item	Location where item is reported
**TITLE**			
Title	1	Identify the report as a systematic review.	Page 1 (Title)
**ABSTRACT**			
Abstract	2	See the PRISMA 2020 for Abstracts checklist.	Page 2
**INTRODUCTION**			
Rationale	3	Describe the rationale for the review in the context of existing knowledge.	Page 3
Objectives	4	Provide an explicit statement of the objective(s) or question(s) the review addresses.	Page 3-4
**METHODS**			
Eligibility criteria	5	Specify the inclusion and exclusion criteria for the review and how studies were grouped for the syntheses.	Page 5-6
Information sources	6	Specify all databases, registers, websites, organisations, reference lists and other sources searched or consulted to identify studies. Specify the date when each source was last searched or consulted.	Page 5
Search strategy	7	Present the full search strategies for all databases, registers and websites, including any filters and limits used.	Supplementary
Selection process	8	Specify the methods used to decide whether a study met the inclusion criteria of the review, including how many reviewers screened each record and each report retrieved, whether they worked independently, and if applicable, details of automation tools used in the process.	Page 5-6
Data collection process	9	Specify the methods used to collect data from reports, including how many reviewers collected data from each report, whether they worked independently, any processes for obtaining or confirming data from study investigators, and if applicable, details of automation tools used in the process.	Page 6
Data items	10a	List and define all outcomes for which data were sought. Specify whether all results that were compatible with each outcome domain in each study were sought (e.g. for all measures, time points, analyses), and if not, the methods used to decide which results to collect.	Page 6
	10b	List and define all other variables for which data were sought (e.g. participant and intervention characteristics, funding sources). Describe any assumptions made about any missing or unclear information.	Page 6-7
Study risk of bias assessment	11	Specify the methods used to assess risk of bias in the included studies, including details of the tool(s) used, how many reviewers assessed each study and whether they worked independently, and if applicable, details of automation tools used in the process.	Page 6
Effect measures	12	Specify for each outcome the effect measure(s) (e.g. risk ratio, mean difference) used in the synthesis or presentation of results.	Page 6-7
Synthesis methods	13a	Describe the processes used to decide which studies were eligible for each synthesis (e.g. tabulating the study intervention characteristics and comparing against the planned groups for each synthesis (item #5)).	Page 6-7
	13b	Describe any methods required to prepare the data for presentation or synthesis, such as handling of missing summary statistics, or data conversions.	Page 6-7
	13c	Describe any methods used to tabulate or visually display results of individual studies and syntheses.	Page 6
	13d	Describe any methods used to synthesize results and provide a rationale for the choice(s). If meta-analysis was performed, describe	Page 6-7

Page Section and Topic	Item #	Checklist item	Location where item is reported
		the model(s), method(s) to identify the presence and extent of statistical heterogeneity, and software package(s) used.	
	13e	Describe any methods used to explore possible causes of heterogeneity among study results (e.g. subgroup analysis, meta-regression).	Page 6
	13f	Describe any sensitivity analyses conducted to assess robustness of the synthesized results.	Page 6-8
Reporting bias assessment	14	Describe any methods used to assess risk of bias due to missing results in a synthesis (arising from reporting biases).	Page 6
Certainty assessment	15	Describe any methods used to assess certainty (or confidence) in the body of evidence for an outcome.	Page 6-8
**RESULTS**			
Study selection	16a	Describe the results of the search and selection process, from the number of records identified in the search to the number of studies included in the review, ideally using a flow diagram.	Page 9
	16b	Cite studies that might appear to meet the inclusion criteria, but which were excluded, and explain why they were excluded.	Page 9
Study characteristics	17	Cite each included study and present its characteristics.	Page 9 and 28
Risk of bias in studies	18	Present assessments of risk of bias for each included study.	Page 9-10
Results of individual studies	19	For all outcomes, present, for each study: (a) summary statistics for each group (where appropriate) and (b) an effect estimate and its precision (e.g. confidence/credible interval), ideally using structured tables or plots.	Page 9-12
Results of syntheses	20a	For each synthesis, briefly summarise the characteristics and risk of bias among contributing studies.	Page 9-12
	20b	Present results of all statistical syntheses conducted. If meta-analysis was done, present for each the summary estimate and its precision (e.g. confidence/credible interval) and measures of statistical heterogeneity. If comparing groups, describe the direction of the effect.	Page 10-11
	20c	Present results of all investigations of possible causes of heterogeneity among study results.	Page 10
	20d	Present results of all sensitivity analyses conducted to assess the robustness of the synthesized results.	Page 10-12
Reporting biases	21	Present assessments of risk of bias due to missing results (arising from reporting biases) for each synthesis assessed.	Page 9-10
Certainty of evidence	22	Present assessments of certainty (or confidence) in the body of evidence for each outcome assessed.	Page 9-10
**DISCUSSION**			
Discussion	23a	Provide a general interpretation of the results in the context of other evidence.	Page 13
	23b	Discuss any limitations of the evidence included in the review.	Page 15-16
	23c	Discuss any limitations of the review processes used.	Page 15-16
	23d	Discuss implications of the results for practice, policy, and future research.	Page 13-16
**OTHER INFORMATION**			

Page Section and Topic	Item #	Checklist item	Location where item is reported
Registration and protocol	24a	Provide registration information for the review, including register name and registration number, or state that the review was not registered.	Page 5
	24b	Indicate where the review protocol can be accessed, or state that a protocol was not prepared.	Page 5
	24c	Describe and explain any amendments to information provided at registration or in the protocol.	Page 5
Support	25	Describe sources of financial or non-financial support for the review, and the role of the funders or sponsors in the review.	Page 18
Competing interests	26	Declare any competing interests of review authors.	Page 18
Availability of data, code and other materials	27	Report which of the following are publicly available and where they can be found: template data collection forms; data extracted from included studies; data used for all analyses; analytic code; any other materials used in the review.	Page 18

Table S2. Search strategy for Ovid MEDLINE.

Search: **("pulsed field ablation" OR PFA OR electroporation OR "Non-thermal ablation" OR Electroablation) AND ("high power short-duration" OR HPSD OR "high power short duration" OR vHPSD OR "very high power short duration" OR "radiofrequency ablation" OR radiofrequency) AND ("atrial fibrillation" OR AF OR Afib OR "persistent atrial fibrillation" OR "paroxysmal atrial fibrillation" OR persAF)** (((("pulse"[MeSH Terms] OR "pulse"[All Fields] OR "heart rate"[MeSH Terms] OR ("heart"[All Fields] AND "rate"[All Fields]) OR "heart rate"[All Fields] OR "pulses"[All Fields] OR "pulse s"[All Fields] OR "pulsed"[All Fields] OR "pulsing"[All Fields]) AND ("field"[All Fields] OR "field s"[All Fields] OR "fields"[All Fields]) AND ("ablate"[All Fields] OR "ablated"[All Fields] OR "ablates"[All Fields] OR "ablating"[All Fields] OR "ablation"[All Fields] OR "ablational"[All Fields] OR "ablations"[All Fields])) OR "PFA"[All Fields] OR ("electroporate"[All Fields] OR "electroporated"[All Fields] OR "electroporates"[All Fields] OR "electroporating"[All Fields] OR "electroporation"[MeSH Terms] OR "electroporation"[All Fields] OR "electroporations"[All Fields] OR "electroporative"[All Fields] OR "electroporator"[All Fields] OR "electroporators"[All Fields] OR "electropore"[All Fields] OR "electropores"[All Fields]) OR ("Non-thermal"[All Fields] AND ("ablate"[All Fields] OR "ablated"[All Fields] OR "ablates"[All Fields] OR "ablating"[All Fields] OR "ablation"[All Fields] OR "ablational"[All Fields] OR "ablations"[All Fields])) OR "Electroablation"[All Fields]) AND (("high"[All Fields] AND ("power, psychological"[MeSH Terms] OR ("power"[All Fields] AND "psychological"[All Fields]) OR "psychological power"[All Fields] OR "power"[All Fields] OR "powered"[All Fields] OR "powers"[All Fields] OR "powering"[All Fields]) AND "short-duration"[All Fields]) OR "HPSD"[All Fields] OR ("high"[All Fields] AND ("power, psychological"[MeSH Terms] OR ("power"[All Fields] AND "psychological"[All Fields]) OR "psychological power"[All Fields] OR "power"[All Fields] OR "powered"[All Fields] OR "powers"[All Fields] OR "powering"[All Fields]) AND ("short"[All Fields] OR "shorts"[All Fields]) AND ("duration"[All Fields] OR "durations"[All Fields])) OR "vHPSD"[All Fields] OR ("high"[All Fields] AND ("power, psychological"[MeSH Terms] OR ("power"[All Fields] AND "psychological"[All Fields]) OR "psychological power"[All Fields] OR "power"[All Fields] OR

"powered"[All Fields] OR "powers"[All Fields] OR "powering"[All Fields]) AND ("short"[All Fields] OR "shorts"[All Fields]) AND ("duration"[All Fields] OR "durations"[All Fields])) OR ("radiofrequency ablation"[MeSH Terms] OR ("radiofrequency"[All Fields] AND "ablation"[All Fields]) OR "radiofrequency ablation"[All Fields]) OR ("radiofrequencies"[All Fields] OR "radiofrequency"[All Fields] OR "radiofrequent"[All Fields])) AND ("atrial fibrillation"[MeSH Terms] OR ("atrial"[All Fields] AND "fibrillation"[All Fields]) OR "atrial fibrillation"[All Fields] OR "AF"[All Fields] OR ("atrial fibrillation"[MeSH Terms] OR ("atrial"[All Fields] AND "fibrillation"[All Fields]) OR "atrial fibrillation"[All Fields] OR "afib"[All Fields]) OR ("atrial fibrillation"[MeSH Terms] OR ("atrial"[All Fields] AND "fibrillation"[All Fields]) OR "atrial fibrillation"[All Fields] OR ("persistent"[All Fields] AND "atrial"[All Fields] AND "fibrillation"[All Fields]) OR "persistent atrial fibrillation"[All Fields]) OR ("atrial fibrillation"[MeSH Terms] OR ("atrial"[All Fields] AND "fibrillation"[All Fields]) OR "atrial fibrillation"[All Fields] OR ("paroxysmal"[All Fields] AND "atrial"[All Fields] AND "fibrillation"[All Fields]) OR "paroxysmal atrial fibrillation"[All Fields]) OR "persAF"[All Fields])

Translations

pulsed: "pulse"[MeSH Terms] OR "pulse"[All Fields] OR "heart rate"[MeSH Terms] OR ("heart"[All Fields] AND "rate"[All Fields]) OR "heart rate"[All Fields] OR "pulses"[All Fields] OR "pulse's"[All Fields] OR "pulsed"[All Fields] OR "pulsing"[All Fields]

field: "field"[All Fields] OR "field's"[All Fields] OR "fields"[All Fields]

ablation: "ablate"[All Fields] OR "ablated"[All Fields] OR "ablates"[All Fields] OR "ablating"[All Fields] OR "ablation"[All Fields] OR "ablational"[All Fields] OR "ablations"[All Fields]

electroporation: "electroporate"[All Fields] OR "electroporated"[All Fields] OR "electroporates"[All Fields] OR "electroporating"[All Fields] OR "electroporation"[MeSH Terms] OR "electroporation"[All Fields] OR "electroporations"[All Fields] OR "electroporative"[All Fields] OR "electroporator"[All Fields] OR "electroporators"[All Fields] OR "electropore"[All Fields] OR "electropores"[All Fields]

ablation: "ablate"[All Fields] OR "ablated"[All Fields] OR "ablates"[All Fields] OR "ablating"[All Fields] OR "ablation"[All Fields] OR "ablational"[All Fields] OR "ablations"[All Fields]
power: "power, psychological"[MeSH Terms] OR ("power"[All Fields] AND "psychological"[All Fields]) OR "psychological power"[All Fields] OR "power"[All Fields] OR "powered"[All Fields] OR "powers"[All Fields] OR "powering"[All Fields]
power: "power, psychological"[MeSH Terms] OR ("power"[All Fields] AND "psychological"[All Fields]) OR "psychological power"[All Fields] OR "power"[All Fields] OR "powered"[All Fields] OR "powers"[All Fields] OR "powering"[All Fields]
short: "short"[All Fields] OR "shorts"[All Fields]
duration: "duration"[All Fields] OR "durations"[All Fields]
power: "power, psychological"[MeSH Terms] OR ("power"[All Fields] AND "psychological"[All Fields]) OR "psychological power"[All Fields] OR "power"[All Fields] OR "powered"[All Fields] OR "powers"[All Fields] OR "powering"[All Fields]
short: "short"[All Fields] OR "shorts"[All Fields]
duration: "duration"[All Fields] OR "durations"[All Fields]
radiofrequency ablation: "radiofrequency ablation"[MeSH Terms] OR ("radiofrequency"[All Fields] AND "ablation"[All Fields]) OR "radiofrequency ablation"[All Fields]
radiofrequency: "radiofrequencies"[All Fields] OR "radiofrequency"[All Fields] OR "radiofrequent"[All Fields]
atrial fibrillation: "atrial fibrillation"[MeSH Terms] OR ("atrial"[All Fields] AND "fibrillation"[All Fields]) OR "atrial fibrillation"[All Fields]
Afib: "atrial fibrillation"[MeSH Terms] OR ("atrial"[All Fields] AND "fibrillation"[All Fields]) OR "atrial fibrillation"[All Fields] OR "afib"[All Fields]
persistent atrial fibrillation: "atrial fibrillation"[MeSH Terms] OR ("atrial"[All Fields] AND "fibrillation"[All Fields]) OR "atrial fibrillation"[All Fields] OR ("persistent"[All Fields] AND "atrial"[All Fields] AND "fibrillation"[All Fields]) OR "persistent atrial fibrillation"[All Fields]
paroxysmal atrial fibrillation: "atrial fibrillation"[MeSH Terms] OR ("atrial"[All Fields] AND "fibrillation"[All Fields]) OR "atrial fibrillation"[All Fields] OR ("paroxysmal"[All Fields] AND "atrial"[All Fields] AND "fibrillation"[All Fields]) OR "paroxysmal atrial fibrillation"[All Fields]

Warnings

(pulsed field ablation OR PFA OR electroporation OR Non-thermal ablation OR Electroablation) AND (high power short-duration OR HPSD OR high power short duration OR vHPSD OR **very** high power short duration OR radiofrequency ablation OR radiofrequency) AND (atrial fibrillation OR AF OR Afib OR persistent atrial fibrillation OR paroxysmal atrial fibrillation OR persAF)

Stop word: very

Table S3. Demographics of included patients from the selected studies

Study	Age (mean±SD)		Male (%)		LVEF (Mean±SD)		BMI (Mean±SD)		PAF (%)		PersAF (%)	
	HPSD	PFA	HPSD	PFA	HPSD	PFA	HPSD	PFA	HPSD	PFA	HPSD	PFA
Abdelrazik, 2024	63±2.7	62.8±3.2	70	66	NR	NR	NR	NR	41	27	9	23
Badertscher, 2023	Total: 65±10		NR	NR	NR	NR	NR	NR	Total: 55.7		NR	NR
Cardinali, 2024	NR	NR	NR	NR	NR	NR	NR	NR	0	0	69.6	30.4
Popa, 2023	63.5±10.6	61.6±11.4	54.4	46.2	58.7±6	58.5±2.7	26.3±4.1	26.7±4.9	63.9	36.1	0	0
Russo, 2024	66.5±2.2	64.5±1.9	64.9	62.6	58.8±0.8	58.3±1.2	25.2±0.4	25.1±0.3	34.2	33.6	15.8	16.4
Reinsch, 2024	67.5±2.9	67±2.6	53.1	56.7	NR	NR	27.5±1.1	27.3±0.9	51	49	0	0
Soubh, 2024	73±7	67±10	47	71	47±14	52±8	27±6	28±6	43	48	57	52
Wörmann, 2023	67±12	67±13	40.4	33.3	56±9	56±6	27±4	28±5	14.9	14.9	35.1	35.1

Study	DM (%)		HP (%)		CHA2DS2-VASc score (Mean±SD)		Coronary Artery Disease (%)	
	HPSD	PFA	HPSD	PFA	HPSD	PFA	HPSD	PFA
Abdelrazik, 2024	NR	NR	NR	NR	NR	NR	NR	NR
Badertscher, 2023	NR	NR	NR	NR	NR	NR	NR	NR
Cardinali, 2024	NR	NR	NR	NR	3±1.5	2.5±1.3	NR	NR
Popa, 2023	6.5	2.9	56.5	40	2±1.6	1.7±1.7	NR	NR
Russo, 2024	20	22.8	62	61	2±0.4	2±0.4	13	14
Reinsch, 2024	13	11	67	66	2±0.4	2.25±0.5	10	9
Soubh, 2024	13	15	83	83	4±1.7	4±1.6	43	17
Wörmann, 2023	14	16	60	65	3	3	19	25

BMI: Body mass index; DM: diabetes; HP: hypertension; HPSD: high-power short duration; LVEF: left ventricular ejection fraction; NR: not reported; PAF: paroxysmal atrial fibrillation; PersAF: persistent atrial fibrillation; PFA: pulsed field ablation; SD: standard deviation.

Table S4. Assessment of risk of bias using the Newcastle Ottawa Scale.

STUDY	SELECTION	COMPARABILITY	OUTCOME/ EXPOSURE
Abdelrazik, 2024	***	**	***
Badertscher, 2023	****	*	***
Cardinali, 2024	***	*	**
Popa, 2023	****	**	***
Reinsch, 2024	**	*	*
Russo, 2024	****	**	*
Soubh, 2024	****	*	**
Wörmann, 2023	****	**	**

Figure S1. Schoenfeld residuals plot assessing the proportional hazards assumption for freedom from AF recurrence

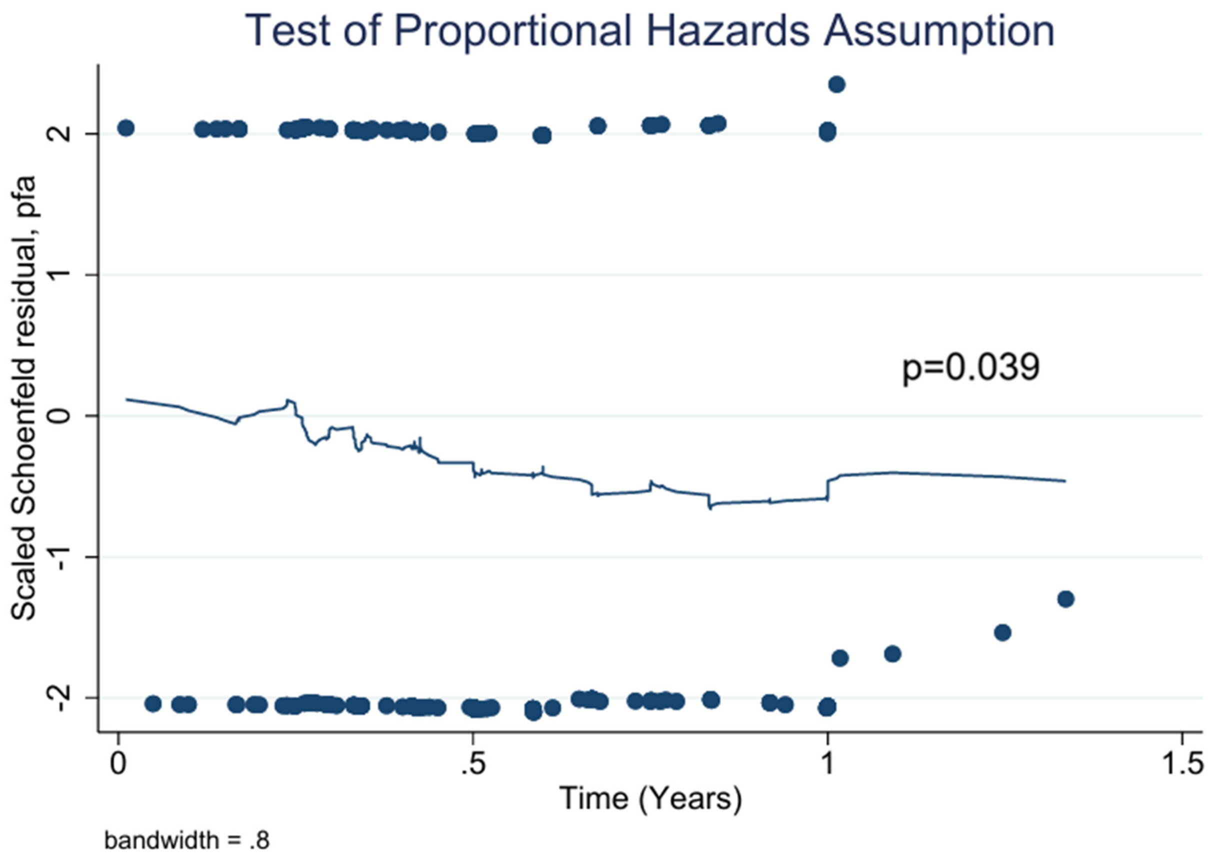

Figure S2. Two-stage random-effects meta-analysis of freedom from AF recurrence

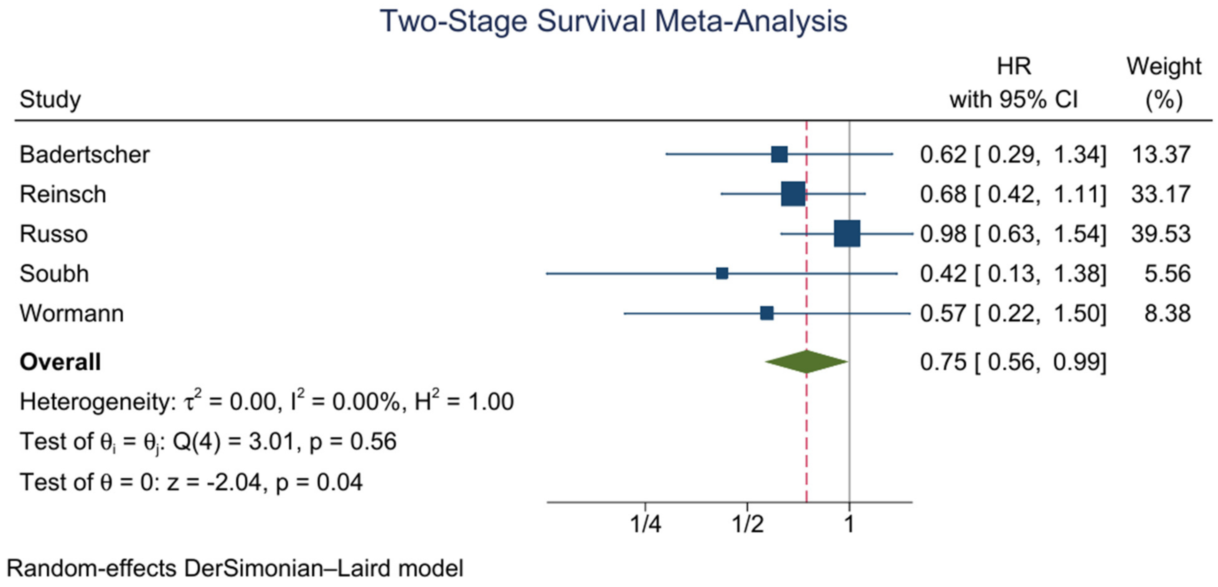

Figure S3. Leave-one-out analysis for procedure duration.

Study	P-value	Tau2	Tau	I2	Mean Difference IV, Random, 95% CI
Omitting Abdelrazik, 2024	< 0.01	104.379	10.2166	87%	34.05 [25.64, 42.46]
Omitting Badertscher, 2023	< 0.01	147.632	12.1504	88%	39.18 [29.28, 49.09]
Omitting Cardinali, 2024	< 0.01	174.515	13.2104	90%	36.56 [26.15, 46.98]
Omitting Popa, 2023	< 0.01	169.657	13.0253	90%	38.45 [28.01, 48.89]
Omitting Reinsch, 2024	< 0.01	103.880	10.1922	75%	33.94 [25.37, 42.51]
Omitting Russo, 2024	< 0.01	170.561	13.0599	89%	38.54 [27.99, 49.09]
Omitting Soubh, 2024	< 0.01	182.236	13.4995	90%	37.42 [26.70, 48.14]
Omitting Wörmann, 2023	< 0.01	176.741	13.2944	90%	38.23 [27.55, 48.91]
**Total (95% CI)**	**< 0.01**	**154.002**	**12.4098**	**89%**	**37.05 [27.69, 46.41]**

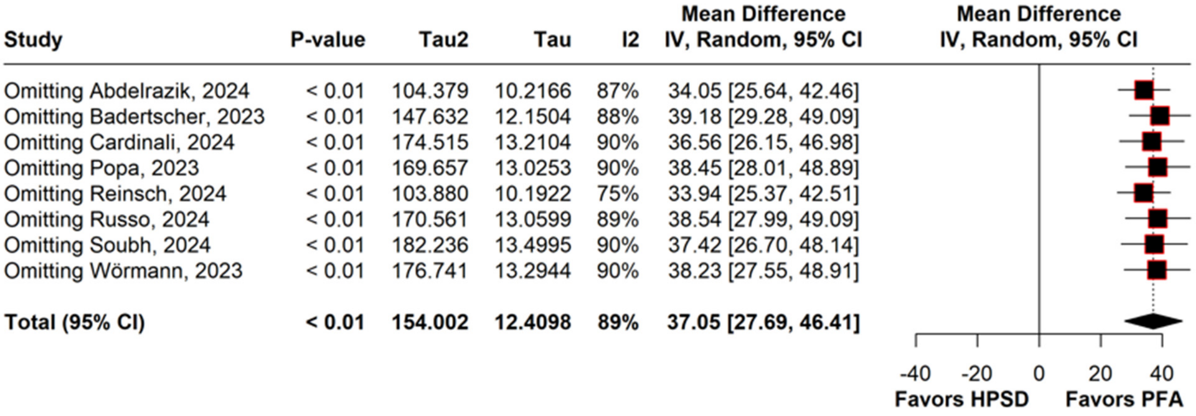

Figure S4. Leave-one-out analysis for fluoroscopy duration.

Study	P-value	Tau2	Tau	I2	Mean Difference IV, Random, 95% CI
Omitting Abdelrazik, 2024	< 0.01	12.163	3.4876	96%	-8.43 [-11.17, -5.70]
Omitting Badertscher, 2023	< 0.01	15.226	3.9021	96%	-8.80 [-11.86, -5.74]
Omitting Cardinali, 2024	< 0.01	15.035	3.8776	96%	-9.15 [-12.13, -6.17]
Omitting Reinsch, 2024	< 0.01	12.648	3.5564	93%	-8.44 [-11.27, -5.62]
Omitting Popa, 2023	< 0.01	14.452	3.8016	96%	-8.71 [-11.67, -5.75]
Omitting Russo, 2024	< 0.01	15.582	3.9474	96%	-9.21 [-12.31, -6.12]
Omitting Soubh, 2024	< 0.01	12.711	3.5653	96%	-9.62 [-12.43, -6.81]
Omitting Wörmann, 2023	< 0.01	7.798	2.7925	92%	-9.98 [-12.27, -7.70]
**Total (95% CI)**	**< 0.01**	**13.251**	**3.6402**	**96%**	**-9.04 [-11.71, -6.37]**

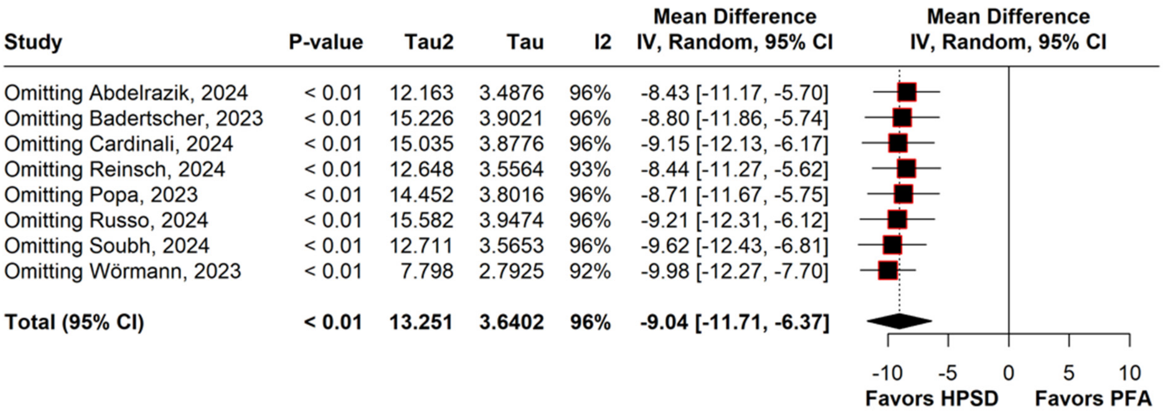

Supplement: Supplementary file 1 [file jcdd-13-00181-s001.zip › jcdd-4187177-supplementary.pdf]
